# Entering an era of dynamic structural biology*…*

**DOI:** 10.1186/s12915-018-0533-4

**Published:** 2018-05-31

**Authors:** Allen M. Orville

**Affiliations:** Diamond Light Source, Research Complex at Harwell, and University of Oxford, Oxfordshire, OX11 0DE UK

## Abstract

A recent paper in *BMC Biology* presents a general method for mix-and-inject serial crystallography, to facilitate the visualization of enzyme intermediates via time-resolved serial femtosecond crystallography (tr-SFX). They apply their method to resolve in near atomic detail the cleavage and inactivation of the antibiotic ceftriaxone by a β-lactamase enzyme from *Mycobacterium tuberculosis*. Their work demonstrates the general applicability of time-resolved crystallography, from which dynamic structures, at atomic resolution, can be obtained.

See research article: https://bmcbiol.biomedcentral.com/articles/10.1186/s12915-018-0524-5.

## Commentary

We are currently experiencing parallel step-changes in structural biology. On the one hand, X-ray crystallography and cryo-EM are essential tools for structural biologists, wherein the raw data are almost always derived from samples held at 100 K. In particular, the rapid advances in cryo-EM are clearly having a large impact across the community [1]. But, life is dynamic and function is not compatible with the cryogenic conditions. Incidentally and on the other hand, X-ray free electron lasers (XFELs) offer new opportunities because their unparalleled intensity makes it possible for even submicron size crystals to yield high quality structures. To this end, serial femtosecond crystallography (SFX) methods that exploit slurries of microcrystals and the XFEL fs pulses are most often conducted at ambient temperature. Therefore, SFX methods are well suited to couple structural biology with functional dynamics [2].

Advanced synchrotron beamlines that produce high-flux (10^13^–10^14^ photons/s), microfocus beamlines (≤ 5 × 5 μm^2^) are either in operation now or under development at nearly all synchrotron facilities [3] (Table 1)—these beamlines are also pushing the development of serial data collection methods that exploit smaller samples and room temperature. For example, the VMXi beamline at Diamond can deploy a double multilayer monochromator (DMM) to deliver > 10^14^ photons/s at 13 keV with an energy band-pass (ΔE/E) of ~ 5 × 10^− 3^ (pink beam). Such a narrow polychromatic source delivers more X-ray photons on the sample and yields more Bragg reflections at the detector per exposure compared to traditional monochromatic diffraction. Pink beam diffraction experiments are also not as complex as Laue methods that exploit broader polychromatic sources. VMXi is designed for room-temperature measurements where the 300 kGy dose limit will be reached within ~ 30–100 μs using the unattenuated, focused beam. The dose limit is typically described by a general loss of diffraction quality of the crystal, whereas local changes in the macromolecule (such as reduction of a metal centre) often experience X-ray-induced photophysical perturbations with orders of magnitude less dose. Moreover, specific alterations of a metal centre or chromophore often result in changes to their spectroscopic signal(s) and also provide a means to follow and characterize mechanistically relevant perturbations to the sample. These types of beamlines also typically field the newest and fastest detectors operating at 750 Hz or more. Therefore, synchrotron characteristics enable time-resolved MX studies with macromolecular catalytic systems operating in the μs/ms and longer time regime [4].

**Table 1:** Examples of synchrotron and XFEL sources for serial crystallography methods

	Diamond light source			XFELs (~ 50 fs pulse duration)				
Beamline	I04	I24	VMXi	Eu.XFEL	LCLS-II	SACLA	SwissFEL	PAL XFEL
Photons/time	5 × 10^11^ (s^−1^)	3 × 10^12^ (s^− 1^)	> 10^14 a^ (s^− 1^)	~ 10^12^ (pulse^− 1^)	≥ 10^12^ (pulse^− 1^)	2 × 10^11^ (pulse^− 1^)	~ 7 × 10^11^ (pulse^− 1^)	2 × 10^11^ (pulse^− 1^)
Beam size (μm^2^)	~ 10 × 5	~ 5 × 5	≤ 5 × 5	< 1–10 × 10	< 1–3 × 3	1.3 × 1.3	< 3 × 3	< 3 × 3
Detector/**pulse (Hz)**	25–100 (750) ^b^	25–100	750 ^b^	**27,000** ^c^	**120–** **10** ^**6** d^	**60**	**100**	**60**

^a^Using a double multilayer monochromator (double crystal monochromator DCM > 2 × 10^12^ ph/s)

^b^Dectris Eiger 4 M or region of interest within Eiger 16 M

^c^2700 pulses in a 600-μs train (4.5 MHz) at 10 Hz

^d^LCLS-II (~ 2020, soft X-rays at 10^6^, hard at 120 Hz) and LCLS-II-HE (~ 2022, soft and hard X-rays at 10^6^ Hz)

**Fig. 1:**
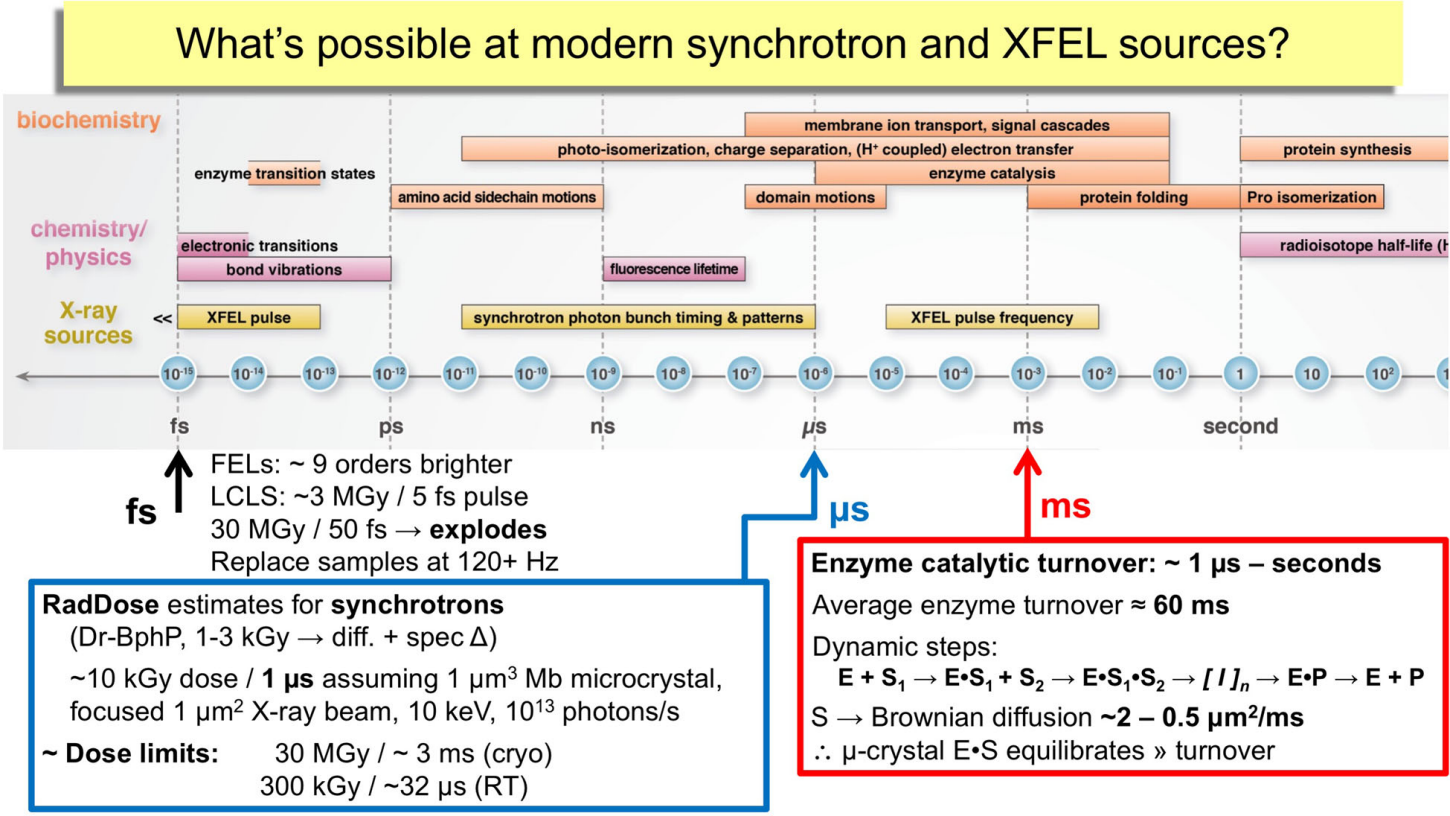
A comparison of the time scales at which different X-ray sources can be applied, and the phenomena they might be used to observe. Biochemical processes span orders of magnitude in time, with faster process linked to electron transfer and photoisomerization, and slower events linked to conformational changes. Substrate diffusion into micron-sized crystals is faster than the average enzyme turnover time. The X-ray dose and time required to elicit a spectroscopic change and an X-ray diffraction pattern to high resolution at a modern synchrotron are illustrated with *Deinococcus radiodurans* phytochrome (*Dr-BphP*) and myoglobin (*Mb*)

For observing shorter time scales, smaller crystals, and samples that are radiation sensitive, a number of XFELs are available (Table 1). For instance, the LCLS and European XFEL deliver 10^12^–10^13^ photons/50 fs pulse, in a ≤ 5 × 5 μm^2^ size beam, with 5 to 1 × 10^− 3^ band-pass in SASE mode. The Cornell–SLAC Pixel Array Detector (CSPAD) matches the 120 Hz at LCLS, and the Adaptive Gain Integrating Pixel Detector (AGIPD) at the European XFEL can collect 3250 images per second from the 27,000 Hz pulses. LCLS-II and LCLS-II-HE plan to deliver MHz X-ray pulse frequencies and new detectors are anticipated to leverage these capabilities. Consequently, synchrotron sources complement XFEL sources and together they provide a wide range of experimental conditions (Fig. 1). For samples that do not contain radiation-sensitive centres and catalyse reaction cycles that are relatively slow, then synchrotrons provide the appropriate capabilities. When reaction cycles include redox active chromophores and/or very reactive, short-lived intermediates, such as Fe(IV)=O oxoferryl, then XFEL facilities are more appropriate. In either case, it is important that the sample is at physiological temperature if functionally relevant dynamics are to be included in the study.

Intense XFEL pulses destroy crystalline biological samples [5]. Fortunately, the structural information is encoded in the photons of the X-ray diffraction pattern travelling at the speed of light, whereas the sample explosion happens at roughly the speed of sound. Serial femtosecond crystallography therefore requires that a new sample is in place for each X-ray pulse. This is often achieved by using flow-focusing gas dynamic virtual nozzles (ff-GDVN) that create small liquid jets. To clear the debris from the interaction region and replenish new sample, the jets must continually inject new sample into the interaction region at ~ 10–30 m/s at the LCLS delivering 120 Hz pulses. Much faster jets are needed to fully exploit the MHz repetition rates of the European XFEL and the LCLS-II. These considerations also place size constraints on the crystals in the sample and, consequently, slurries of microcrystals are ideal. Furthermore, a critical step to producing samples for SFX methods is very often observed as a shower of microcrystals during an initial crystallization trial of most macromolecular samples. Flow-focusing GDVN methods also provide opportunities to add substrates via the outer, flow-focusing fluid, directly to slurries of microcrystals within the central focused jet [6]. This strategy is similar to a continuous flow transient kinetic scheme. Micron-size crystals will equilibrate via diffusion with small molecule substrates on the μs time scale, which is much faster than the 60-ms average turnover time of enzymes. If electrostatic interactions between the enzyme and the substrate(s) are present, then one could anticipate even faster equilibration times. Therefore, time-resolved SFX methods (tr-SFX) that produce high quality electron density maps are generalizable. An area of particular importance is the creation of new tools and strategies to manipulate microcrystal samples that may provide a means to correlate enzyme kinetics, spectroscopy, and time-resolved structural biology.

The paper by Olmos et al. [7] in this issue is among the first to demonstrate that ff-GDVN mixing jets and tr-SFX methods can be used to observe transient intermediates of enzymes engaged in catalysis. They use the phrase ‘mix-and-inject serial crystallography’ (MISC) and demonstrate the capability with BlaC, a beta-lactamase from *Mycobacterium tuberculosis*, catalysing the ring cleavage of ceftriaxone, a so-called third-generation cephalosporin antibiotic. They collected tr-SFX datasets at 30, 100, 500 and 2000 ms timepoints after mixing with substrate at the Linac Coherent Light Source at SLAC National Accelerator Laboratory in California, USA. The resulting electron density maps reveal features that are modelled as reaction intermediates that build up and go away with reaction time after mixing. Refinements of the atomic models fit to 2.15–2.75 Å resolution maps are used to estimate the relative concentrations of the intermediates at the timepoints and are fit to a kinetic model. Although these results are supported by electron density maps alone, the addition of complementary spectroscopic and catalytic studies from the same sample(s) will become more readily available and will provide important verification of critical reactive intermediates in future studies on a wide range of systems [8].

One of the important results of the study is the clear demonstration that the crystal packing significantly impacts reaction rates. It makes logical sense that a crystal form with a smaller solvent content and narrower intra-lattice channels will not equilibrate with substrates soaked into the lattice as fast as those with larger solvent content and larger channels [9]. Consequently, one should evaluate more than one space group and unit cell packing to increase the likelihood of success for a MISC experiments. This also needs to be balanced against the particular mechanistic question(s) to be addressed, and the resolution of the electron density maps achievable from each crystal form. The manuscript also shows that single turnover reaction schemes rapidly produce a high product concentration that can subsequently inhibit enzyme reactions as they move from transient to steady-state kinetic regimes.

This year, about 700,000 people worldwide will die from drug-resistant infections. Professor Dame Sally Davies, the chief medical officer in the UK, said recently that “without action we risk infection related mortality returning to pre-antibiotic levels by the mid-21st century”. An independent review on antimicrobial resistance, chaired by macroeconomist Jim O’Neill, suggests that by 2050 mortality rates could reach 10 million people per year or about 1 out of every 1000 people [10]. The status quo in structure–function analysis in structural biology is to use many separate samples, from which different types of data are collected under different conditions that may be far from physiological. To this end, structure-based drug discovery and fragment-based screening are major strategies to bring new drugs to market. And yet, most new drugs fail because they lack efficacy. These strategies typically rely upon ground-state crystal structures determined at 100 K. Therefore, an emerging alternative that de-risks the path towards new therapeutics is to study the entire reaction cycle at physiological temperature. Indeed, with temporal, dynamic and functional data linked to atomic models, it is likely that new mechanistic insights will suggest novel strategies to mediate or inhibit function. For instance, one might target a transient state revealed by tr-SFX methods that is not sufficiently populated or observed by traditional ligand soak and cryo-cool approaches.

New X-ray sources create exciting new opportunities and as a result we are entering an era of *dynamic structural biology.* This is as much a concept as a set of tools to collect as much data as possible, from every sample and X-ray pulse, and enables one to create atomic resolution ‘movies’ of macromolecules engaged in catalysis. Thus, time-resolved crystallography, a longstanding frontier challenge for the field, is achievable with serial methods at XFELs and at advanced synchrotron beamlines.
